# The Rural Community-Based Occupational Therapy Model (Rural CB-OT): Community-Codesigned Solution to support Aging in Place in Rural Underserved Areas

**DOI:** 10.21203/rs.3.rs-10933834/v1

**Published:** 2026-09-08

**Authors:** Tiago S Jesus, Kaleb Masterson, Lisa Juckett, Cory Cronin

**Affiliations:** The Ohio State University, School of Health and Rehabilitation Sciences; The Ohio State University; The Ohio State University; Ohio University

**Keywords:** Occupational Therapy, Rehabilitation, Rural Health Services, Appalachian Region, Aging in Place

## Abstract

**Importance:**

Rural older populations experience barriers to accessing rehabilitation, occupational therapy (OT), and aging-in-place services. Access may be enhanced by service models designed by underserved communities to fit local resources and priorities.

**Objective:**

To describe the process and outcomes of a community-led codesign in rural Appalachia, Ohio, to co-create a locally tailored, community-based rehabilitation service model that supports aging in place.

**Design:**

Community-based participatory research involving a regional Steering Committee that selected a target county and codesigners. Codesigners participated in two workshops guided by a double-diamond framework and informed by rehabilitation-access maps, user personas, and evidence-based program options. Steering Committee feedback refined the model and helped identify transferable and adaptable implementation components.

**Setting:**

Medically-underserved rural Appalachia, Ohio.

**Participants:**

Seven committee members and five county-level codesigners representing public health, aging services, and community organizations.

**Outcomes and Measures:**

Community-generated rehabilitation service model and its core and adaptable implementation components.

**Results:**

Codesigners developed the Rural Community-Based Occupational Therapy (Rural CB-OT) concept, comprising six core components: community-based OT, trusted community hub, multiple service-delivery modes, task sharing, local health-system and community partnerships, and community-academic partnerships. Participants also identified context-sensitive implementation options, including team structure, population scope, hub selection, and funding.

**Conclusions and Relevance:**

Rural CB-OT offers a preliminary framework for expanding community-based OT in underserved rural communities while illustrating how evidence-informed, community-led codesign can generate locally owned rehabilitation solutions with transferable core components and adaptable implementation features. Future research should focus on further coproduction, implementation, and evaluation of Rural CB-OT and the underlying codesign approach.

**Plain-Language Summary:**

Community stakeholders in rural Appalachia used local data and evidence-informed resources to codesign Rural CB-OT, a community-based occupational therapy model intended to improve access to rehabilitation and support aging in place. Rather than selecting a single program, participants developed a flexible service framework that could support multiple rehabilitation needs while adapting to local resources and priorities.

## Introduction

Rural individuals with disabilities, including older adults, experience substantial barriers to healthcare access, which contribute to poorer health outcomes (Mesmar et al., 2025). These inequities are often driven by workforce shortages, lower service availability, long travel distances, and financial constraints that limit access to care (Baker et al., 2024; Siconolfi et al., 2019). Rural populations experience higher rates disability than urban populations and are disproportionately older. For example, 19.9% of rural residents were aged 65 years or older compared with 16.0% of urban residents (United States Census Bureau, 2023). Adjusted for age and sociodemographics, rural residents are 9% more likely to report a disability, 24% more likely to report three or more disabilities, and 18% more likely to report long-term disability (Sage et al., 2019; Zhao et al., 2019). Some under-resourced rural communities, including parts of Appalachia and the Deep South, experience even higher age-adjusted disability rates alongside poorer care access and adverse social determinants of health (Arena et al., 2024). As rural populations age and disability prevalence increases, access to rehabilitation becomes increasingly important for supporting independence, participation, and aging in place.

National studies show that rural residents with disabilities are less likely to receive postacute rehabilitation and, when they do, receive less frequent and less intensive services than urban residents (Jesus et al., 2026; Kosar et al., 2020). Geographic disparities are particularly pronounced for home- and community-based rehabilitation, where rural residents exhibit lower utilization despite greater need (Ayres et al., 2026; Jesus et al., 2026; Quigley et al., 2022). Recent changes in national payment policies may place rural residents at additional risk of reduced access to needed rehabilitation, thereby contributing to poorer outcomes and lower opportunities for aging in place (Loomer et al., 2023; Mroz et al., 2020).

Occupational Therapy (OT) is well positioned to promote community health and aging-in-place services with older populations (Kafel et al., 2026; Stotz et al., 2025). Growing evidence base supports community- and home-based interventions, many OT-led, including multicomponent fall-prevention programs, CAPABLE, and dementia caregiver interventions (Best Programs for Caregiving, 2026; National Council on Aging, 2026; Rahmati et al., 2023). These programs improve function, caregiver outcomes, and quality of life, while often reducing healthcare utilization and costs (Elliott & Leland, 2018; Jutkowitz et al., 2025). However, rural OT delivery faces workforce, reimbursement, recruitment, retention, transportation, and sustainability challenges that may limit implementation and scale-up in rural underserved communities. (Ayres, 2026; Hayes et al., 2023). Moreover, most evidence-based interventions are condition-specific initiatives requiring dedicated workforce, infrastructure, implementation strategies, or funding. In resource-constrained rural communities, sustaining multiple parallel programs may be challenging, contributing to fragmented services and unmet needs (Wilkinson et al., 2022).

Addressing rural rehabilitation access gaps may require integrated, locally adapted service-delivery models that align with local resources and workforce capacity while integrating multiple evidence-based interventions within a single service framework (Hauck et al., 2019; Wilkinson et al., 2022). Integrated approaches such as the Program of All-Inclusive Care for the Elderly (PACE) provide a coordinated service delivery; however, their workforce and infrastructure requirements have limited implementation in many rural underserved communities (National Advisory Committee on Rural Health and Human Services (NACRHHS), 2023). Consequently, underserved rural communities may benefit from horizontally organized service-delivery models capable of integrating multiple evidence-based interventions within a common service structure, aligning with local priorities, available resource, and workforce capacity (Wilkinson et al., 2022).

Community-led codesign offers a promising approach to developing rehabilitation and aging-in-place services that reflect the needs, priorities, and implementation realities of underserved rural communities. Compared with traditional top-down approaches, community-led codesign positions local stakeholders as leaders in identifying unmet needs and designing locally tailored services, potentially improving relevance, implementability, and sustainability (Aguilar-Gaxiola et al., 2022; Morley et al., 2024; Ramanadhan et al., 2024). Despite growing interest in participatory approaches within OT (Kang et al., 2024), we are unaware of published studies describing a structured, evidence-informed, community-led codesign process used to develop an integrated OT, rehabilitation, and aging-in-place service model tailored to underserved rural communities.

Against this backdrop, this study describes the process and outcomes of a community-led codesign undertaken in rural Appalachia, Ohio, to co-create a community-based rehabilitation service model that supports aging in place in medically underserved rural communities.

## METHODS

### Design:

This community-based participatory research study used a data- and evidence-informed, community-led codesign approach to develop an innovative, community-based, locally tailored rehabilitation service-delivery model to support aging in place in rural, medically underserved Appalachian Ohio. This paper describes the codesign approach and tools used to facilitate model development and presents the resulting co-created service-delivery model centered on a novel Rural Community-Based Occupational Therapy (Rural CB-OT) role.

### Ethics:

The Ohio State University Institutional Review Board approved the study (STUDY20250099). All participants provided written informed consent, including for disclosing their names/backgrounds.

### Community-Based Codesign Overview

Figure 1 overviews the community-based codesign process. It involved seven regional Steering Committee (SC) members (Table 1) from across Appalachian Ohio who served as co-researchers throughout. SC members contributed to county selection, participant recruitment, and refinement of the final service-delivery model.

Drawing on their local knowledge and interactive rehabilitation-access maps developed by the researchers (Step 1), SC members selected a highly underserved county and identified local stakeholders to participate in county-level codesign workshops (Step 2). Concurrently, the research team developed a codesign toolkit, including a menu of evidence-based program options to inform solution development (Step 3). County-level, in-person workshops were then conducted using the Double Diamond framework (Sevillano-Garayoa et al., 2026) to facilitate co-creation of a novel, locally tailored, community-based rehabilitation service-delivery model supporting aging in place (Step 4). Following model development, SC members provided feedback on its operationalization, implementability, and transferability to other rural contexts (Step 5).

This paper reports the codesign process (Steps 1-5) and resulting service-delivery model. Process evaluation the examining participants’ experiences with the codesign approach and tools (Step 6) will be reported elsewhere.

### Mapping Data and County Selection (Steps 1 and 2):

To support evidence-informed site selection, the research team translated previously analyzed public-domain data (Jesus et al., 2026) into interactive county-level maps of access to rehabilitation across Ohio. Specifically, risk-adjusted county-level postacute rehabilitation utilization data, particularly from home health agencies, were synthesized into a county-level rehabilitation utilization index (Jesus, 2026a). This index was visualized alongside complementary need and access-risk indices, capturing population-level rehabilitation needs (e.g., disability prevalence and disabling health conditions) and contextual barriers to access (e.g., transportation limitations, rurality, and social vulnerability) (Jesus, 2026a). Together, these data were translated into interactive maps designed to support Steering Committee deliberations regarding local rehabilitation access gaps.

The maps are publicly available and include descriptions of data sources, computational methods, and navigation instructions (Jesus, 2026a). For illustrative purposes, Figure 2 presents snapshots of the gap-index choropleth maps, including a statewide overview and a zoomed view of Appalachian Ohio.

Steering Committee members reviewed and interacted with the maps before and during two virtually facilitated meetings. Following an overview of the map methodology, features, and findings by the principal investigator (TJ), members engaged in a deliberative dialogue (Oelke et al., 2016) combining mapped data with their local knowledge and lived experience to identify a community for the county-level codesign. Meigs County was ultimately selected based on its high rehabilitation-access gap index, Steering Committee validation of its underserved status, and the perceived availability of local stakeholders to participate in the codesign. Steering Committee members subsequently leveraged their professional and community networks, including snowball recruitment through local organizational leaders, to identify and invite county-level codesign participants. Table 1 presents the final group of five Meigs County codesigners, who primarily represented public health, community health, and senior-service organizations.

### Building Codesign Tools (Step 3)

To support community-engaged codesign, the research team developed a toolkit to enable an evidence-informed codesign. Community-based codesign involves more than bringing stakeholders together; it requires providing community codesigners with accessible, actionable information through digestible data- and evidence-translation tools that support their collective deliberation and decision-making (Jull et al., 2017; Morley et al., 2024). Accordingly, the researchers developed a codesign toolkit comprising three complementary tools aligned with the two stages/diamonds of the Double Diamond framework: (a) detailed maps and (b) user personas were developed to support collective problem definition and situational assessment (first diamond), and (c) a menu of evidence-based options to support the codesign of new service delivery solutions (second diamond). **Supplementary Appendix 1** provides the full toolkit used to facilitate the codesign, synthesized below.

#### a) Detailed Maps:

To support situational assessment during the first diamond of the Double Diamond framework, the research team developed two detailed interactive maps by disaggregating the broader access indicators previously used by the Steering Committee for county selection. Specifically, aggregated measures were decomposed into more granular domain-level (e.g., county-level normalized rates of need associated with neurological conditions) and indicator-level metrics (e.g., county-level normalized stroke prevalence rates). These maps are publicly available online (Jesus, 2026b, 2026c). By translating complex population-level data into accessible visual formats, the maps supported evidence-informed deliberation regarding local needs, access barriers, and opportunities for improvement prior to codesigning locally-tailored service delivery solutions.

#### b) User Personas:

Four fictional personas represented individuals recovering from stroke, recovering from hip fracture and at risk of falls, living with multiple chronic conditions and disabilities, and providing informal caregiving to an older adult with Alzheimer’s disease. The personas reflected common support needs, service-use patterns, and access barriers experienced by older adults with disabilities in underserved rural communities. To support collective problem definition and situational assessment during the first diamond of the Double Diamond framework, the personas translated diverse user experiences into accessible narratives that fostered empathy and consideration of the rehabilitation needs, preferences, and challenges of different community members. The mixed-methods process used to develop the personas (e.g., integrative literature synthesis, qualitative interviews, and stakeholder feedback) is reported elsewhere (Masterson et al., 2026). The personas are available in Supplementary Appendix 1 (pp. 8-12)

#### C) Menu of Evidence-Based Options:

To support solution development during the second diamond of the Double Diamond framework, the research team developed a catalog (Menu) of evidence-based program options that translated evidence from diverse sources into accessible service-delivery options, workforce innovations, and implementation considerations that could be deliberated, adapted, combined, or rejected by community codesigners. The Menu was developed using knowledge-translation and toolkit-development (Hempel et al., 2019; Janvrin et al., 2023; Juckett et al., 2022) drawing on established evidence-based program repositories (Best Programs for Caregiving, 2026; National Council on Aging, 2026; Rural Information Hub, 2025) and recent reviews and guidelines (Ayres et al., 2026; Canneaux et al., 2026; Kafel et al., 2026; Nicholson et al., 2024; Rahmati et al., 2023).

The Menu of Options (**Supplementary Appendix 1**, pp. 13-28; **Supplementary Appendix 2** provides the accompanying slide-based version also used during codesign workshops) consisted of four categories of evidence-based programs aligned with the personas, five service-delivery innovations (e.g., telerehabilitation; rehabilitation in primary care), five workforce innovations for underserved rural areas (e.g., task-sharing with community providers), two integrated-care innovations, and an appendix of local funding opportunities and regional supports.

Within the Menu, the categories of evidence-based programs aligned with the four personas were: (1) an OT-based home-transition program after stroke (Bollinger et al., 2024); (2) fall-prevention programs (e.g., exercise-based and multi-component) (Elliott & Leland, 2018; Nicholson et al., 2024) (3) the CAPABLE program (Rahmati et al., 2023); and (4) three evidence-based OT-led programs for dementia caregivers: COPE, TAP, and Skill2Care^®^ (Best Programs for Caregiving, 2022a, 2022b). For each option, the Menu summarized its description, evidence base, outcomes, implementation examples, cost considerations, and rural adaptations.

Although the Menu often included OT-led approaches, some options did not rely on occupational therapy (e.g., the Otago Exercise Program). Codesigners were not encouraged or expected to include OT in the final service model.

### County-level codesign (Step 4)

Following recruitment, county-level codesigners attended two 2.5-hour in-person workshops conducted a few days apart and guided by a double-diamond codesign framework (Sevillano-Garayoa et al., 2026). The principal investigator (TJ) led the workshop facilitation, supported by another researcher (KM).

In the first workshop, codesigners focused on collective problem definition and situational assessment using a Strengths-Weaknesses-Opportunities-Threats (SWOT) framework (Jesus et al., 2023) applied to the context of Meigs County. Deliberations were informed by the detailed maps, user personas, and codesigners’ own community-based experiences. Using the SWOT findings, participants then identified and initially selected locally suitable options from the Menu to inform the emerging service model.

Between workshops, the researchers synthesized participants’ input into an initial visual representation of the program concept (**Supplementary Appendix 3**), drawing on workshop sketches, field notes, and audio recordings.

The second workshop focused on solution development and refinement. Codesigners reviewed the preliminary program concept, further specified its components and operations, considered potential funding sources, and identified implementation requirements, facilitators, barriers, and adaptation strategies.

Consistent with principles of community-led codesign for health-system strengthening (Aguilar-Gaxiola et al., 2022; Lindblom et al., 2021), researchers acted as facilitators rather than solution designers. Their role was limited to introducing activities and tools, providing technical clarification, synthesizing participant input, asking clarifying questions, and ensuring all voices were heard.

### Steering Committee Input (Step 5), Final Synthesis, and Validation.

Following county-level codesign, SC members provided feedback on the codesigned model through a virtual 1-hour focus group based on the researchers’ synthesis of the workshop outputs and facilitated by the principal investigator (TJ), who also facilitated the codesign. Input was sought on model operationalization, implementation barriers, facilitators and implementation strategies, and transferability to other underserved rural areas of Ohio using an IRB-approved semi-structured focus-group guide. Resultant data were analyzed using conventional qualitative content analysis (Hsieh & Shannon, 2005). The same two investigators who attended the county-level workshops independently reviewed the focus-group transcript and notes, coded statements related to model components, implementation considerations, and transferability, and reconciled interpretations through discussion. Findings informed refinement of the final visual model and accompanying narrative. Reporting followed the Consolidated Criteria for Reporting Qualitative Research (COREQ) checklist (Tong et al., 2007).

Consistent with implementation science guidance on intervention design (Damschroder et al., 2009; Stokes et al., 2018), the final synthesis distinguished between core components, considered essential to the model’s function, and adaptable components, which reflected local preferences and contextual considerations, which may be modified to fit other communities’ circumstances. The resulting, researcher-synthesized and refined model and accompanying narrative were subsequently validated by both SC members and county-level codesigners.

## RESULTS

The county-level codesign in Meigs County, Appalachian Ohio, produced the Rural Community-Based Occupational Therapy (Rural CB-OT) model, a community-led approach to addressing aging-in-place rehabilitation gaps in medically underserved rural communities. The model comprised six core components alongside several context-sensitive, adaptable implementation options.

### 1. Rural CB-OT Model

Figure 3 presents the Rural CB-OT model. The model comprises six interrelated core components viewed as essential for implementation: (1) a community-based OT, (2) a trusted community hub, (3) four complementary service-delivery modes, (4) task sharing with community-based personnel, (5) local health-system and community partnerships, and (6) regional community-academic partnerships.

#### 1.1. Community-Based Occupational Therapist

Codesigners identified a community-based OT as the central model feature. Participants recognized that many community assets, organizations, and personnel already existed locally; however, they identified the absence of community-based rehabilitation expertise as a critical gap limiting the local delivery of evidence-based rehabilitation and aging-in-place programs. Consequently, a licensed OT was viewed as the key enabling professional capable of bringing rehabilitation expertise directly into the community. Embedded within a trusted community organization serving older adults, the OT would facilitate service delivery, supervision, and task sharing across the model’s components while connecting existing health, social, and community-based resources. Core responsibilities included assessment, care planning, individualized intervention, education, advocacy, care coordination, and supervision of community-based personnel.

#### 1.2. Community Hub

Codesigners identified a trusted community hub as a needed anchor for service delivery and community engagement. Rather than creating a new healthcare-based service, participants emphasized embedding rehabilitation services within community organizations already trusted by local older adults. The hub was viewed as critical for improving access, fostering trust, supporting prevention, and integrating rehabilitation services within existing community resources:

“*Older adults [here] are so concerned with us coming in and telling them they can no longer live at home, and we’re going to put them in a facility […] they donť want you connected to any kind of nursing facility*.” (SCM3)

In Meigs County, codesigners identified the Blakeslee Center, which houses the Meigs County Council on Aging, a senior center, and other community- and home-based programs, as a suitable community hub.

#### 1.3. Four Service Delivery Modes

Codesigners envisioned four complementary service-delivery modes that could be flexibly combined according to individual needs, preferences, and contextual constraints.

##### Hub-Based Services.

The community hub was viewed as an accessible entry point for rehabilitation and preventive services delivered in a familiar community setting. Suggested OT activities included screening, assessment, health education, individual and group interventions, and participation-focused programming, including group-based and preventive components of evidence-based programs such as fall prevention. Locating services within existing community organizations was also viewed as strengthening coordination with community-based resources.

##### Home-Based Services.

Home-based services were considered essential for addressing environmental barriers and supporting individuals with more complex needs. Prioritized interventions included home assessment, environmental modification, assistive technology, caregiver support, and individualized care planning. This service mode aligned closely with evidence-based programs such as CAPABLE, post-stroke transition interventions, dementia caregiver interventions, and home-based components of fall-prevention programs (Supplementary Appendix 1).

##### Tele-Supported Services.

These services were viewed as a strategy for overcoming transportation barriers, workforce shortages, and geographic isolation. Codesigners considered telehealth valuable primarily for screening, monitoring, and follow-up rather than primary assessment or intervention because user personas and stakeholder discussions highlighted barriers related to broadband access, digital literacy, and preferences for retaining an in-person component. Consequently, tele-supported services were envisioned as a community-mediated follow-up strategy, whereby OTs provide remote support while community-based personnel already conducting home visits facilitate virtual interactions and implementation of recommendations.

##### Community-Wide Services.

Beyond individual service provision, codesigners emphasized addressing community-level determinants of participation through collaboration with health and community partners. Suggested activities included health promotion, occupational justice initiatives, accessibility improvements, environmental modification efforts, and disability-inclusive community initiatives.

#### 1.4. Task Sharing

Across all four service-delivery modes, codesigners emphasized task sharing as a key implementation strategy. Task sharing, defined as delegation of selected activities to trained community-based personnel under OT supervision, was viewed as a means of extending service reach, increasing follow-up capacity, addressing workforce shortages, and reducing implementation costs. Existing community health workers, volunteers, aides, assistants, and other trusted personnel were identified as important partners. The approach aligned with existing local initiatives and was strongly endorsed by SC members:

“I like the idea of the community health workers. I think that we need to carve out specific places for community health workers because with the healthcare worker shortage, it’s only going to get worse.”(SCM6)

“I definitely think that local… community health workers can assist to help get people connected to services and also to advocate for additional services within their service area.”(SCM7)

#### 1.5. Local Health-System and Community Partnerships

Codesigners emphasized that the community-based OT should operate in partnership with existing health-system and community organizations. Partnerships with healthcare providers, managed care organizations, public health agencies, aging-service organizations, and community-based organizations were viewed as essential for referrals, care coordination, service integration, and connecting older adults with existing supports. Referral generation was viewed as particularly important for implementation success:

“One of the forever questions that we have… is getting this older adult population to see value in accessing the services and not waiting until there’s a crisis… I’m very intrigued by this moving on, and somehow…. getting the referrals and getting those connections.” (SCM3)

“I definitely think that local health departments… can assist to help get people connected to services and also to advocate for additional services…. The Long Term Care Ombudsman as another possible source… The 211 referral system… might also be able to assist… The local Area Agencies on Aging might be able to assist with possible case management services or information and referral.”(SCM7)

#### 1.6. Regional Community-Academic Partnerships

Regional community-academic partnerships were identified as important sources of model development, implementation, specialist, and workforce support. Codesigners viewed these partnerships as facilitating model refinement, implementation planning, training, program evaluation, quality improvement, and tele-mentoring through hub-and-spoke approaches such as Project ECHO (Supplementary Appendix 1). Partnerships were also viewed as supporting sustainable rural rehabilitation workforce development. Nevertheless, codesigners emphasized that external partners should support rather than direct implementation, thereby preserving community ownership and responsiveness to local priorities.

### 2. Adaptable Periphery: Context-Sensitive Implementation Options

Participants emphasized that communities differ in resources, organizational structures, partnerships, and funding environments. Consequently, although the six core components were viewed as foundational and potentially transferable beyond Meigs County, implementation features were expected to vary by context. Team structure, catchment area, population scope, hub selection, and funding mechanisms were identified as elements requiring local tailoring and coproduction.

#### 2.1. Team Structure and Catchment Area

Codesigners in Meigs County favored a locally embedded model centered on a single OT serving one community as a base case. This configuration was viewed as maximizing community trust, ownership, and responsiveness while requiring modest implementation resources.

Participants nevertheless recognized that other contexts may support broader implementation structures. Larger populations, expanded geographic coverage, or future growth could justify incorporation of OT assistants, physical therapists, or larger interprofessional teams. Serving multiple neighboring counties was also discussed, albeit not preferred by Meigs County stakeholders.

These discussions highlighted a trade-off between service capacity and community embeddedness. Although larger service areas could increase reach and economies of scale, they were also viewed as requiring greater transportation, coordination, and administrative resources while potentially weakening local relationships and community ownership. Overall, team structure and catchment area were viewed as context-dependent implementation decisions.

#### 2.2. Population Scope

Although local codesigners developed Rural CB-OT to address aging-in-place needs among older adults, expansion to other populations with unmet rehabilitation needs (e.g., children with developmental disabilities) was discussed. While a broader scope could increase reach, economies of scale, and reimbursement opportunities, codesigners felt it would add complexity to early implementation and increase onboarding demands for a single OT.

For Meigs County, expansion beyond older adults was generally viewed as a later-stage possibility following successful implementation and demonstration of value, or as part of future team expansion. A SC member also suggested extending services to younger older adults:

“[we could] start focusing on those younger older adults to… [keep] functioning younger.” (SCM3)

#### 2.3. Community Hub Selection

Codesigners consistently identified a trusted community-based hub as an essential model component and service-delivery anchor. Senior centers emerged as the preferred setting because of their established role and engagement with older adults. Although Meigs County codesigners selected the local senior center as the preferred hub, SC members considered this approach scalable across the region.

“I think [senior centers in the region would] definitely be interested in something like this. [Another senior center] is set up well for [replication]”(SCM6)

Although senior centers were the preferred local example, SC members identified community health centers, federally qualified health centers, and community-based service organizations as potentially suitable hubs or hub partners in other rural underserved settings.

“We could engage… a community health center or Federally Qualified Health Center…. with a Person-Centered Medical Home model.”(SCM1)

#### 2.4. Funding & Sustainability

Codesigners and SC members identified funding and sustainability as critical implementation considerations. Although research or pilot funding was viewed as important for initial developing and testing, participants emphasized the need to coproduce financing models capable of supporting routine operation.

Rather than advocating for a single funding mechanism, participants discussed multiple revenue streams, including Medicaid-funded home- and community-based services, Medicare caregiver and care-management billing opportunities, primary care partnerships, local aging and health initiatives, and managed care contracts. For example, contracts with health plans participating in Ohio’s Next Generation MyCare program for dual-eligible beneficiaries were identified as one potential financing strategy. Such arrangements were viewed favorably because capitated payment structures of those programs incentivize prevention, aging in place, care coordination, and avoidance of costly institutional care.

More broadly, participants viewed the model as aligned with emerging value-based payment approaches and emphasized the importance of demonstrating return on investment to potential funders and partners.

“*I see this alignment with the trend for a value-based health care… [and] it is cheaper to prevent the second stroke than to treat it*.”(SC1)

Existing but underutilized community funding opportunities were also noted.

“*We sit on Title III evidence-based money that is available for evidence-based programs … but we can get no one to take that money*.”(SC6)

Collectively, these discussions positioned sustainability not as a single funding solution but as a locally negotiated, context-specific process requiring collaboration among community organizations, health systems, funders, and academic partners.

## Discussion

This study reports the process and outcomes of a data-informed community-based codesign undertaken in rural Appalachia, Ohio, to address rehabilitation access gaps affecting aging in place in medically underserved communities. Through a community-led, evidence-informed process supported by mapped data, user personas, and evidence-based options, participants developed the Rural CB-OT concept comprising six interconnected core components and several context-sensitive implementation adaptations. Importantly, Rural CB-OT should be understood as a community-generated program concept rather than a validated service model. Consistent with contemporary codesign frameworks (Aguilar-Gaxiola et al., 2022; Pearce et al., 2020), this study represents a co-ideation phase; further coproduction, implementation, and evaluation are needed before feasibility, effectiveness, or sustainability can be established. Nevertheless, the findings demonstrate how underserved communities, when enabled, can generate locally owned rehabilitation solutions through structured, evidence-informed, community-led processes that incorporate implementation fit and local adaptation from the outset. Both the codesign process and Rural CB-OT may have broader relevance because of their emphasis on local adaptation and the distinction between core and adaptable components.

The resulting model reflects a systems-strengthening response to rural rehabilitation and aging-in-place challenges. The codesigned model combines hub-based, home-based, tele-supported, and community-wide activities supported by a community-based OT anchored within a trusted community organization. Participants did not respond to rehabilitation access gaps by selecting a single evidence-based program. Instead, they designed a shared workforce and service-delivery infrastructure capable of supporting multiple interventions and aging-in-place needs. This horizontal approach may be particularly relevant in underserved rural communities where limited workforce capacity, travel burden, and organizational resources make implementation of multiple condition-specific programs difficult to sustain (Hauck et al., 2019; Wilkinson et al., 2022). Participants also emphasized the importance of a trusted community hub, suggesting that trust, familiarity, and community legitimacy may be as important for implementation and acceptability beyond literature-identified barriers such as transportation, provider shortages, and geographic isolation (Ayres, 2026; Mesmar et al., 2025).

Beyond the resulting model, the study contributes to translational science by demonstrating how evidence can be mobilized to support community-led service design. Rather than translating predefined interventions into a community, stakeholders were provided with evidence-informed resources and retained the agency to select, combine, adapt, or reject options based on perceived local relevance and implementability. One example was the addition of community-based mediators (e.g., community health workers, volunteers, and home-based providers) to support telerehabilitation, despite its evidence base, because stakeholders identified implementation barriers related to rurality and local older populations’ preferences (Ayres, 2026; Hepburn et al., 2025; Rettinger & Kuhn, 2023). Evidence therefore informed rather than prescribed the resulting model. This approach aligns with integrated knowledge translation, community-engaged translational science, and participatory implementation science, which position communities as active co-creators of knowledge rather than recipients of externally developed interventions (Jull et al., 2017; Ramanadhan et al., 2024; Rhodes et al., 2021). It also moves beyond the traditional use of community participants merely as testers, informants, or focus-group participants that remains common in community-engaged OT research (Kang et al., 2024).

Beyond underserved rural communities overall, such empowerment may be particularly important in rural Appalachia, a region with a history of economic and health inequities and externally driven interventions (Cronin, 2023; Keefe, 2009). These experiences have sometimes contributed to skepticism toward healthcare, research, and development initiatives perceived as originating outside the community (Keefe, 2009). Community-enabled approaches emphasizing local ownership, capacity building, and shared decision making may therefore be especially important for fostering trust, legitimacy, and long-term sustainability of new service models (Hatfield et al., 2026; Rhodes et al., 2021), including community-generated approaches such as Rural CB-OT.

Aligned with implementation-science guidance (Damschroder et al., 2009; Stokes et al., 2018), Rural CB-OT distinguishes between core and adaptable components from the co-ideation phase. The six core components may represent transferable functions, whereas their operationalization will likely vary across communities. Consequently, Rural CB-OT should be viewed not as a context-bound solution or implementation-ready blueprint, but as a framework for local adaptation and future codesign efforts. Communities facing similar challenges may adapt the Rural CB-OT framework and/or use the described process to develop their own context-sensitive solutions.

A noteworthy finding was that the resulting concept centered on a community-based OT despite OT not being predetermined as part of the solution. Participants recognized that many community assets, organizations, and personnel already existed locally but identified community-based, skilled rehabilitation expertise as a critical missing resource. Consequently, the OT was positioned as a cross-cutting, enabling professional responsible for introducing rehabilitation expertise into the community and connecting existing assets through supervision, coordination, and service integration. This aligns with growing calls for OT to contribute more substantially to population health, healthy aging, aging in place, prevention, and health equity through community-focused and primary care roles (Donnelly et al., 2023; Kafel et al., 2026; Reitz et al., 2020). Codesigners envisioned the OT not within a traditional healthcare setting but within a trusted community organization, operating at the interface of health, social, and community sectors and connecting the environments in which people live, age, and participate. Collectively, Rural CB-OT may expand the use of communities as OT clients while addressing broader population health and occupational justice needs (Hyett et al., 2019; Malfitano et al., 2014; Reitz et al., 2020).

Across all service-delivery domains, the model is underpinned by task sharing, workforce enablement, and cross-sector partnerships. Although task sharing may raise concerns regarding professional role substitution (Bhattacharjya et al., 2024), participants viewed it as a strategy for extending access to specialized rehabilitation expertise and leveraging, rather than replacing, the community-based OT role. The OT remained responsible for assessment, oversight, and program development, whereas community personnel extended the reach and continuity of that expertise under OT supervision, consistent with the rehabilitation task-sharing literature in under-resourced settings (Bhattacharjya et al., 2024; Kumurenzi et al., 2023). Sustainability discussions similarly emphasized cross-sector partnerships, blended financing, and value-based care opportunities. Together, these findings position Rural CB-OT as a community ecosystem rather than a standalone service, suggesting that further ecosystem-level coproduction may be needed before implementation and testing.

Several limitations should be considered. The concept was generated within a single rural Appalachian region and county and therefore reflects the priorities and experiences of stakeholders operating within that context. The study focused on codesign of a context-sensitive solution rather than testing or implementation, precluding conclusions regarding feasibility, acceptability, effectiveness, cost-effectiveness, or sustainability. Additional stakeholder groups, including healthcare providers, payers, and community members receiving services, may identify further opportunities and challenges during future coproduction phases. Although community stakeholders generated a shared vision, preliminary implementation architecture, and potential financing pathways, additional phases of coproduction, co-implementation, and co-evaluation remain necessary before implementation readiness can be established (Pearce et al., 2020). Finally, this paper reports on the codesigned solution, not on the stakeholder evaluation of the whole community-engaged process or its tools, which will be reported elsewhere.

Despite these limitations, this study demonstrates the value of data-informed community-based codesign for generating locally relevant rehabilitation solutions in underserved rural communities. Rural CB-OT positions occupational therapy within trusted community settings and leverages workforce enablement, cross-sector partnerships, and multiple service-delivery approaches to support aging in place. Beyond a preliminary service framework, the study contributes a translational process through which communities can use local data, evidence-informed options, and stakeholder deliberation to develop context-sensitive rehabilitation solutions. Future research should focus on continued coproduction, pilot implementation, and evaluation within Meigs County and other underserved rural communities, while examining how Rural CB-OT and the underlying codesign methodology can be adapted to support locally developed rehabilitation solutions.

## Conclusion

This study reports the process and outcomes of a data-informed community-based codesign undertaken in rural Appalachia, Ohio, to address rehabilitation access gaps affecting aging in place. The study demonstrates how underserved communities can be enabled to translate evidence into locally owned rehabilitation solutions through structured, evidence-informed, community-led codesign. Rural CB-OT comprises six potentially transferable core components alongside adaptable implementation features intended for local tailoring. Consequently, the study’s transferable contribution lies both in the Rural CB-OT framework and in the codesign process that generated it. Future research should focus on coproduction, implementation, and evaluation of Rural CB-OT within Meigs County and similar underserved communities, including assessment of feasibility, acceptability, outcomes, costs, and sustainability, while examining how the framework and codesign methodology can support locally developed rehabilitation solutions elsewhere.

## Supplementary Material

This is a list of supplementary files associated with this preprint. Click to download.

- Supfiles.zip

## Figures and Tables

**Figure 1:**
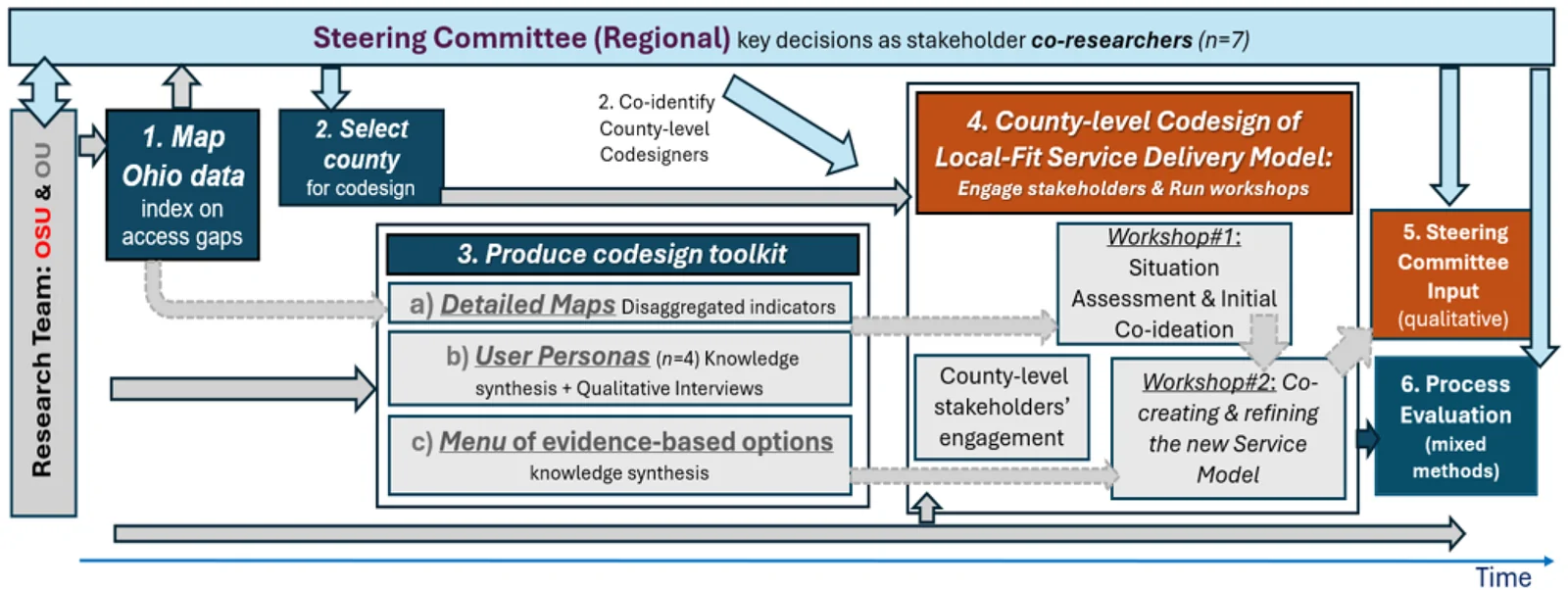
Flowchart of the data-informed, place-based community-based codesign from 8/2025 to 7/2026. OSU: Ohio State University; OU: Ohio University.

**Figure 2:**
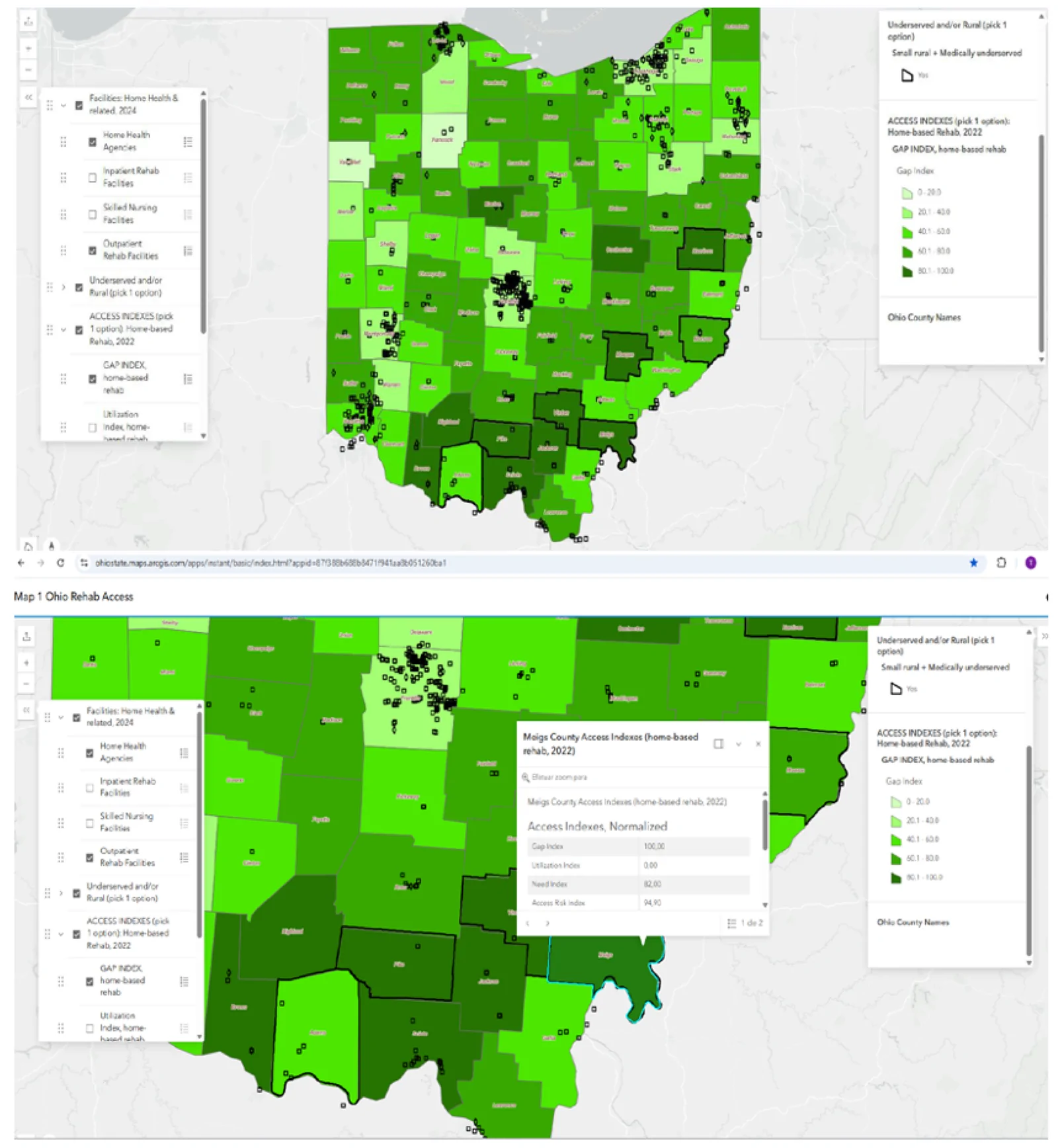
Snapshots of the interactive maps used by Steering Committee members to select a county for the codesign

**Figure 3.**
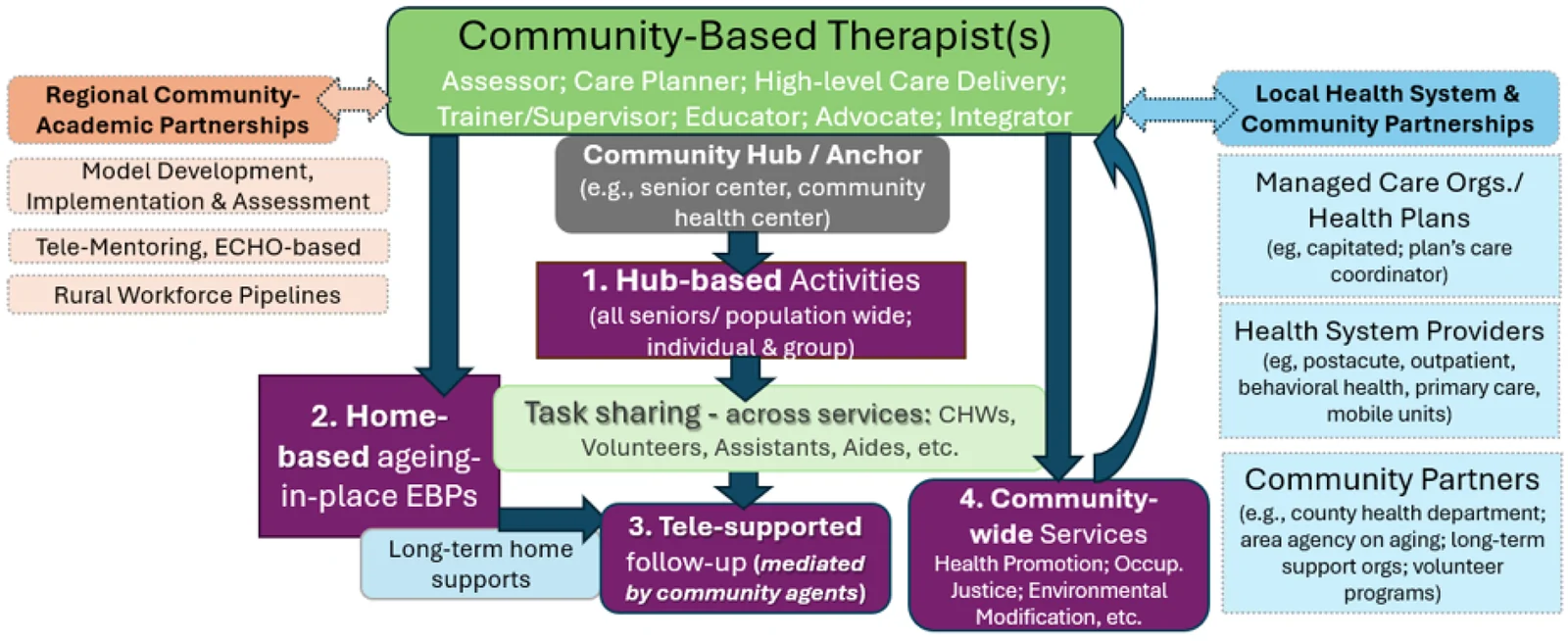
Rural Community-Based Occupational Therapy Model *(RuraiCB-OT):* A Community-led Codesigned Solution to Address Aging-in-Place Service Gaps. **Notes:** Solid arrows: OT service delivery and care processes. Dashed arrows: System interfaces and partnerships

**Table 1:** Participants in the whole community-based codesign, organized by role. All participants were offered hourly-based participant-incentive compensation for the time spent in the role.

Name	Affiliation	Position	Inclusion Rationale
Steering Committee Members			
Jamie Herrmann	Area Agency on Aging District 7(1); Ohio Association of Area Agencies on Aging (2)	Executive Director (1); Board of Directors, member (2)	Oversee senior support services for one of the rural Appalachian regions, Ohio, and knowledgeable of these services statewide.
Jennifer Westfall	Buckeye Hills Regional Council (1); Ohio Association of Area Agencies on Aging (2)	Executive Director (1); Board of Directors, member (2)	Oversees senior support services for one of the rural Appalachian regions, Ohio, and knowledgeable of these services statewide.
Summer Kirby	Ohio Association of Community Health Centers (1); Compass Community Health Clinic (2); Southeast Ohio Community Advisory Board (3)	Chair of the Board of Directors (1); Chief Executive Officer (2); Board Member (3)	Runs a Federally Qualified Health Center in the region, leads the state association of community health centers, and is an experienced advisor on community-engaged research.
Michelle Stumbo	The Ohio State University Extension Offices	Area 24 Leader and Extension Educator	Conducts numerous extension activities in the target areas out of a large state university
Jennifer Kucera	Ohio’s Developmental Disabilities Council & Ohio Olmstead Task Force (1). Center for Disability Empowerment (2). UnitedHealthcare Board: People with Complex Care Needs (3)	Task Force Chair (1); Healthcare Outreach Coordinator (2); Advisory Board Member (3)	Knowledge of long-term and disability-related services, funding, laws, integrated service models and overall supports.
Beverly Stringer	Clinical and Translational Science Institute (OSU) & The Appalachian Translational Research Network	Southeast Ohio Community Engagement Manager	Experienced advisor on community-engaged research in the region
Joe Lemon	Corporation for Ohio Appalachian Development (COAD)	Senior Programs Division Director	Runs the senior programs for a regional organization providing community and volunteering services for the region.
County-level Codesigners - Meigs County, OH			
Rhonda Rathburn	The Blakeslee Center / Meigs Council On Aging	Community Care Coordinator	Knowledgeable of all senior and long-term home & community-based supports provided currently and historically
Marcus Barr	Meigs County Health Department	Health Commissioner	Knowledgeable of population health, health service landscape, funding and Initiative to Improve access to care
Michelle Gorman	Ohio University	Community Health Worker	Representative of regional Community Health Workforce Programs
Elizabeth Jones Ramirez	Integrated Services for Behavioral Health	County-Level Manager, Meigs	County-level representative of a healthcare provider on integrated community-based mental health service
Robin Dewey	Corporation for Ohio Appalachian Development	Senior Companion Coordinator for Meigs among other counties	Local representative of a regional organization providing volunteer support and other companions to seniors
